# Editorial: Ecological Disaster Neuropsychiatry

**DOI:** 10.3389/fpsyt.2021.753243

**Published:** 2021-09-28

**Authors:** Konstantin Loganovsky, Donatella Marazziti, Lars Weisæth

**Affiliations:** ^1^Department of Radiation Psychoneurology, Institute for Clinical Radiology, State Institution “National Research Centre for Radiation Medicine, National Academy of Medical Sciences of Ukraine”, Kyiv, Ukraine; ^2^Section of Psychiatry, Department of Clinical and Experimental Medicine, Pisa, Italy; ^3^Unicamillus – Saint Camillus International University of Health Sciences, Rome, Italy; ^4^Norwegian Centre for Violence and Traumatic Stress Studies, Institute of Clinical Medicine at the University of Oslo, Oslo, Norway

**Keywords:** ecological psychiatry, ecological disaster psychiatry, earthquakes, flood, nuclear accidents

Ecological psychiatry/neuropsychiatry, or ecopsychiatry/econeuropsychiatry, is the application of ecological thought to the study and practice of neuropsychiatry. This term was first used in the late 1970s by the American Psychiatric Association based on mounting evidence of strict relationships between the environment and mental health disorders (1, 2). It is not surprising that thereafter the World Psychiatric Association (WPA) accepted the creation of this novel section while considering the increasingly dramatic challenges following global ecological disasters and emergencies. Indeed, both natural (e.g., climate changes, heat waves, drought, hurricanes, earthquakes) and man-made (e.g., radiation, pollution, chemicals, terrorist attacks, war) emergencies, which are often coupled, may represent unspecific traumatic stressors often resulting in the impairment of well-being and/or physical and mental health deterioration. If it is now evident that psychopathological disorders are the result of the association of individual (genetic) vulnerability coupled with environmental factors acting on epigenetic mechanisms, it is also true that different emergencies may provoke specific neurotoxicity and biological adaptive responses, leading to characteristic neuropsychiatric effects. Therefore, the ensuing neuropsychiatric effects should be considered the final pathways of the combination of stressors and exogenous environmental, physical, chemical, and/or biological factors (3).

Currently, psychiatry has to face a series of increased challenges consequent to unexpected and severe climate change often leading to the migration of populations from afflicted territories toward wealthier societies, as well as epidemics. Accordingly, the latest SARS-COV-2, pandemic with its psychological and neuropsychiatric sequelae, is a clear example of our inability to protect ourselves from climate change or pollution, or from the consequences of human activities disregard for natural habitats and constantly increasing the probability of spillover of viruses from animals to humans (4).

Unfortunately, as psychiatry of disaster has so far been mainly socio-psychologically oriented with an underestimation of the psychopathological consequences, to fill this gap, we organized a special issue of Frontiers collecting seven papers related to neuropsychiatric research dealing with the main ecological emergencies.

In the paper entitled “Prediction and understanding of resilience in Albertan families: longitudinal study of disaster responses (PURLS),” Kingston et al. present a study analyzing the possible factors influencing (or not) the onset of negative effects after the 2013 Alberta Flood. This paper shows the detailed protocol on prediction of resilience in Albertan families, as well as collection procedures, resiliency screening tools, candidate gene identification, genotyping, DNA methylation, and genomic analyses to achieve the research objectives. This study led to novel knowledge on children's health and development, with possible relevance to government policy.

The paper of Morganstein and Ursano, “Ecological disasters and mental health: causes, consequences, and interventions,” is a comprehensive review on ecological disasters and how they are related to global climate changes. The authors underline that predictions can be made on the psychological and behavioral consequences of ecological disasters and their impact on the most vulnerable communities. In addition, they highlight the need for improving information and increasing awareness amongst planners and respondents to reduce negative psychological events and enhance effective preparedness to deal with disaster management.

The paper entitled “The impact of climate change on mental health: a systematic descriptive review,” by Cianconi et al. is a review on the association between climate change and psychiatric disorders. The results show that the effects of climate change can be direct or indirect, short-term or long-term. Acute events can act through mechanisms similar to those of traumatic stress, leading to well-understood psychopathological patterns. On the contrary, the consequences of exposure to prolonged weather-related events encompass different disorders, and could be even transmitted to future generations.

The paper “Disrupted rhythmicity and vegetative functions related to PTSD and gender in earthquake survivors,” by Carmassi, Dell'Oste et al. reports the findings of an original study exploring eventual alterations of biorhythmicity and vegetative functions in a group of high school senior students, exposed to the 2009 L'Aquila earthquake. The results supported the original hypothesis of altered rhythmicity and vegetative functions in survivors, especially in women suffering from PTSD, with respect to those without it. According to the authors, the evaluation of symptoms of impaired rhythmicity and vegetative functions seems essential to reach both the accurate assessment and clinical management of survivors of mass trauma.

In their paper entitled “Sensitivity to climate and weather changes in euthymic bipolar subjects: association with suicide attempts,” Di Nicola et al. investigated the sensitivity to weather and climatic variations in 152 subjects with bipolar disorder and its relationship with lifetime suicide attempts, as compared with 352 healthy control subjects. The results showed that the number of subjects reaching the cut-off for meteorosensitivity/meteoropathy was significantly higher in patients with bipolar disorder. Interestingly, those patients with lifetime suicide attempts obtained higher scores. These findings support the relevance of sensitivity to weather and climate variations and the association of this feature with lifetime suicide attempts at least in bipolar patients.

Loganovsky et al.'s paper entitled “Radiation risk analysis of neuropsychiatric disorders in Ukrainian Chernobyl catastrophe liquidators,” is retrospective-prospective research exploring the possible impact of ionizing radiation on the pathophysiology of neuropsychiatric disorders amongst clean-up workers (liquidators) of the Chernobyl catastrophe The overall findings strongly indicate that liquidators show an increased prevalence of cognitive, affective, and stress-related disorders increasing with radiation dose. Radiation risks are particularly associated with brain damage, organic psychoses, and acute and chronic cerebrovascular pathology.

Another paper from the Italian group dealing with the dramatic aftermath of the L'Aquila 2009 earthquake by Carmassi, Bertelloni et al. entitled “PTSD and suicidal behaviors amongst L'Aquila 2009 earthquake young survivors,” examined the possible relationships between suicidal behaviors and full-blown or partial PTSD in a sample of 475 young survivors, and the specific role of PTSD symptoms on suicidality. The ensuing findings showed that suicidal ideation and suicide attempts were present and more elevated in full-blown PTSD subjects than in those suffering from partial or no PTSD. The results of the present study support and extend previous findings on the role of full-blown PTSD or sub-threshold symptoms in suicidality after a severe earthquake.

Taken together, the papers of this special issue that, in our opinion, is timely and innovative have underlined how massive and deep might theimpact be of ecological disasters on mental health. Last, but not least, we recommend that specialists in psychiatry should be well-educated in ecological psychiatry, given the increasing and unprecedented occurrence of dramatic disasters worldwide. It is also essential that psychiatry takes into account these suggestions in order to be not only a front-line but a sustainable specialty.

## Author Contributions

DM, KL, and LW planned, organized, and selected the papers be included in the special issue. All authors contributed to the article and approved the submitted version.

## Conflict of Interest

The authors declare that the research was conducted in the absence of any commercial or financial relationships that could be construed as a potential conflict of interest.

## Publisher's Note

All claims expressed in this article are solely those of the authors and do not necessarily represent those of their affiliated organizations, or those of the publisher, the editors and the reviewers. Any product that may be evaluated in this article, or claim that may be made by its manufacturer, is not guaranteed or endorsed by the publisher.
